# The immune response after noise damage in the cochlea is characterized by a heterogeneous mix of adaptive and innate immune cells

**DOI:** 10.1038/s41598-020-72181-6

**Published:** 2020-09-16

**Authors:** Vikrant Rai, Megan B. Wood, Hao Feng, Nathan. M. Schabla, Shu Tu, Jian Zuo

**Affiliations:** 1 Department of Biomedical Science, Creighton University School of Medicine, 2500 California Plaza, Omaha, NE 68178 USA; 2 Department of Otolaryngology-Head and Neck Surgery, Johns Hopkins University School of Medicine, 733 N Broadway, Baltimore, MD 21205 USA; 3 Department of Medical Microbiology and Immunology and Flow Cytometry Core, Creighton University School of Medicine, 2500 California Plaza, Omaha, NE 68178 USA; 4 Department of Developmental Neurobiology, St. Jude Children’s Research Hospital, Memphis, TN 38105 USA

**Keywords:** Cochlea, Neuroscience, Neuroimmunology

## Abstract

Cells of the immune system are present in the adult cochlea and respond to damage caused by noise exposure. However, the types of immune cells involved and their locations within the cochlea are unclear. We used flow cytometry and immunostaining to reveal the heterogeneity of the immune cells in the cochlea and validated the presence of immune cell gene expression by analyzing existing single-cell RNA-sequencing (scRNAseq) data. We demonstrate that cell types of both the innate and adaptive immune system are present in the cochlea. In response to noise damage, immune cells increase in number. B, T, NK, and myeloid cells (macrophages and neutrophils) are the predominant immune cells present. Interestingly, immune cells appear to respond to noise damage by infiltrating the organ of Corti. Our studies highlight the need to further understand the role of these immune cells within the cochlea after noise exposure.

## Introduction

The inner ear is not an immune-privileged site^1–3^, and the involvement of the immune system in the noise-induced hearing loss has recently come under increased scrutiny^4,5^. The discovery that CX3CR1+ macrophages were present in the cochlea even before noise damage occurred gave the first indication that the CD45+ immune cells in the ear might have a resident purpose^6,7^. Matern et al.^8^ recently noted that the CD45 + GFI1 + cells in the cochlea at postnatal day 4 (P4) were comprised of T cells, natural killer (NK) cells, and macrophages. Although the immune system is not yet mature at this age^9^, the results of this study indicated that the CD45+ population in the cochlea is heterogeneous, including cells of both the innate (nonspecific, damage-responsive) and adaptive (antigen- specific, responsive) arms of the immune system. The increased recruitment of macrophages in the cochlea after noise damage and the importance of fractalkine signaling in macrophage recruitment has been discussed in the literature^4,10^. Decreased macrophage numbers after disruption of fractalkine signaling results in an increased neuronal pathology^11^. Further, noise damage causes an immune response and CD45+ cell infiltration in response to hair-cell death^6,12–14^. This similar response may be due to the fact that damage- associated molecular patterns (DAMPs) stimulate the pattern recognition receptors^15^. Infiltrating and resident macrophages release inflammatory cytokines such as TNF-α, IL-1β, and IL-6, with peak cell infiltration occurring 4 days after the insult^5,16,17^. Notably, to investigate adaptive immune responses, studies must include later time points after noise damage due to the time needed to generate such a response (approximately 7 days)^18^.

Identifying the types of cells that are present in the adult cochlea in the steady-state and after noise damage may provide additional clues for developing interventions to treat noise- induced damage. In the 1980s, a series of studies performed in guinea pigs showed that an antigen-specific response to keyhole limpet hemocyanin (KLH) could be mounted within the cochlea^19,20^. When KLH was introduced into the cochlea, KLH-specific immune cells could be found in the draining lymph node and spleen^21–23^. Furthermore, KLH-specific antibodies were secreted in the cochlea^20,24^. The results of these studies indicate that antibody-secreting cells, such as B cells, may exist in the cochlea, where they are ready to respond to foreign antigens. These cells have not been reported to be resident or to respond to noise damage in the adult mammalian cochlea; however, our understanding of the adaptive immune response after noise damage would be incomplete without studying both B-cell and T-cell involvement at the appropriate time elapsed.

To elucidate the heterogeneity of immune cells in the unexposed and noise-exposed cochlea, we performed the flow cytometry analysis using markers for various immune cells and then validated the presence of immune cells with immunostaining in the unexposed and noise-exposed cochlea and by detailed analyses of the existing scRNA sequencing data to verify the gene and surface-marker expression of immune cells (innate and adaptive) in the murine adult cochlea. Our findings provide evidence of heterogeneous adaptive- and innate-immune cell responses in the cochlea after noise damage.

## Results

### Flow cytometry showed the heterogeneous immune-cell population in the mouse cochlea

**Figure 1:**
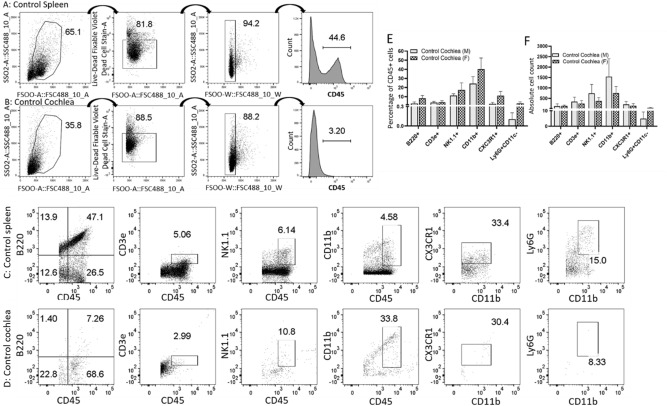
Flow-cytometric analysis of the wild-type adult cochlea indicates the presence of innate and adaptive immune cells. Panels shows the gating strategy for flow-cytometric analysis of the immune cells in control spleen (**A**) and cochleae (**B**) of C57BL/6J mice. A gate was first set from the forward-scatter versus side-scatter dot-plot of all events in the area that corresponded to the size and granularity of lymphocytes using a spleen as a reference. The cells in the lymphocyte gate were then plotted as forward scatter versus Live-Dead stain. The cells in the live gate were then examined in a plot comparing the forward-scatter width and side-scatter area; cells inside the gate were determined to be single cells (singlets). The cells within the singlet gate were then plotted as a histogram of CD45 expression. Live CD45 + cells in the sample were further analyzed for B, T, NK, and myeloid cells and myeloid cells were further gated for macrophages and neutrophils depending on their surface-markers as described in methods section. Representative dot-plots from control C57BL/6 mouse spleen (**C**) and cochlea (**D**) shows various CD45 + immune cells including B, T, NK, and myeloid cells, macrophages and neutrophils with their respective surface expression of B220, CD3e, NK1.1, CD11b, CX3CR1, and Ly6G respectively. The numbers on the dot-plots shows the percentages of cells gated in the corresponding dot-plot. The numbers showed in the plots for B, T, NK, and myeloid cells are percentage of CD45 + cells while the numbers of macrophages and neutrophils are percentage of CD11b + cells, whereas the number for all immune cells in (**E**) are shown as the percentage of CD45 + cells. Panel F shows the absolute number of B cells (CD45 + B220 +), T cells (CD45 + CD3e +), NK cells (CD45 + NK1.1 +), myeloid cells (CD45 + CD11b +), macrophages (CD45 + CD11b + CX3CR1 +), and neutrophils (CD45 + CD11b + Ly6G + CD11c −) in the control cochleae.

We elucidated the heterogeneity of immune cells population in control and noise-exposed cochlea using flow cytometry by developing several independent assays using the C57BL/6J mouse model. We first developed a panel of eight antibodies for our flow- cytometry analysis to identify several major classes of immune cells (Table 1). We set gates using the control spleen sample and then applied the same gates to the cochlea. Briefly, the cells were gated on live via LIVE/DEAD Fixable Violet Dead Cell Stain-lo cells and on single cells. First, we analyzed the total live CD45+ cells from the cochlear cell sample and the live CD45+ cells were further analyzed for other immune cells markers (B220+, CD3e+, NK1.1+, and CD11b+) and the myeloid cells (CD45 + CD11b+) were analyzed for macrophages and neutrophils using respective markers as noted in Table 1. The gating strategy is illustrated in Fig. 1A (control spleen) and B (control cochlea). CD45 + B220 + cells were defined as B cells, and CD45 + CD3e + cells were defined as T cells. CD3e marks both CD4 + and CD8 + cells. CD45 + Nk1.1 + cells were defined as NK cells and these cells CD3e − . It should be noted that NK1.1 is only an NK-cell marker in C57BL/6 J mice and that some CD3e + may co-label for NK1.1^25,26^. CD45 + CD11b + cells were defined as myeloid cells and were split into several populations. Based on their properties in other immune compartments, we defined CD45 + CD11b + CX3CR1 + cells as cochlear macrophages, CD45 + CD11b + Ly6G + CD11c − cells as neutrophils, and CD45 + CD11b + Ly6G + CD11c + cells as monocytes or immature macrophages. There were very few CD45 + CD11b + Ly6G + CD11c + cells. Figures 1C (control spleen) and D (control cochlea) flow dot-plots show the representative average immune cell population (percentage) of 9 C57BL/6 J mice. The myeloid population can be broken down relatively evenly into macrophages, monocytes, and neutrophils. The percentage population and absolute cell counts of the immune cells in control cochleae (Fig. 1E,F) and spleen (Supplementary Fig. 1A,B) suggest a smaller population of immune cells in the cochleae compared to spleen, as expected. Figure 1E shows the percentage of CD45+ cells made up of B220+, CD3e + NK1.1+, CD11b+, CD11b + CX3CR1+, and CD11b + Ly6G + CD11c−. Figure 1F shows the absolute cell numbers of these populations. Figure 1E,F show the relative contributions of each cell type to the overall CD45 + population in the homeostatic cochlea.

**Table 1:** Immune cell phenotypic markers.

Cell type	Positive markers	Negative markers
Immune cell	CD45 +, Live, Singlets	DAPI +, Doublets
B Cell (adaptive immune cell)	CD45 + B220 +	CD3e−, CD11b−
T Cell (adaptive immune cell)	CD45 + CD3e +	B220− CD11b−
Natural killer (NK) cell	CD45 + NK1.1 +	CD3e−
Myeloid cell (innate immune cell)	CD45 + CD11b +	B220− CD3e−
Cochlear macrophage	CD45 + CD11b + CX3CR1 +	
Neutrophil	CD45 + CD11b + Ly6G +	CD11c−
Circulating monocyte	CD45 + CD11b + Ly6G + CD11c +	

Surface markers CD45, B220+, CD3e, NK1.1, CD11b, CX3CR1, and Ly6G were used to sort immune cells, B cells, T cells, NK cells, myeloid cells, macrophages, and neutrophils respectively, by flow cytometry. The detailed strategy has been discussed in the methods section. Table shows the positive and negative markers used to sort the immune cells by flow cytometry.

To verify that our sample was not only made up of immune cells circulating in the blood through the cochlea, we perfused the mice with ice cold PBS with heparin before analyzing the immune cells in the cochlea ^27,28^. Supplementary Fig. 1 shows representative flow cytometry plots (Plots 1C to 1H), average percentages (Supplementary Fig. 1I) and absolute counts (Supplementary Fig. 1J) of the immune cells from perfused cochleae. There were no significant differences in the percentages of each cell type contributing to the immune cell population in the nonperfused and perfused cochleae. The differences in the absolute cell count of the immune cells in the ice-cold perfused cochlea compared to experimental control cochleae (Fig. 1 and Supplementary Fig. 1) suggest that more than half of the immune cells in the cochlea are in the tissue and not merely in the blood passing through the cochlea. We also did the perfusion with warm PBS and compared the results with the control cochlea to control for potential constriction of vessels by ice-cold perfusion. There was no significant difference in the immune cell population between the control and perfused cochlea with warm saline (data not shown).

The expression of several other genes that are specific for B and T cells was observed in the single-cell RNA sequencing data, we analyzed the expression of CD19 and CD24 as additional B-cell markers and CD8a as an additional cytotoxic T-lymphocyte marker in wild-type mice (Data not shown). B and T cells make up most of the cells in the spleen. In the cochlea, most B220 + cells were CD24 +; however, only a small fraction of B220 + cells were CD19 +. CD19 is a maturation marker for B cells, and its expression is lost on plasma cells^29^. It is possible that the small number of CD24 + B220 + CD19—cells are immature bone marrow cells that were included in the sample during tissue preparation. A portion of CD3e + cells was also CD8a +, indicating that some of the T cells in the cochlea may be CD8 + cytotoxic T lymphocytes. From this data, we conclude that the CD45 + cells in the steady-state adult cochlea include both adaptive and innate cell types.

### Immune responses in the cochlea after noise damage

**Figure 2:**
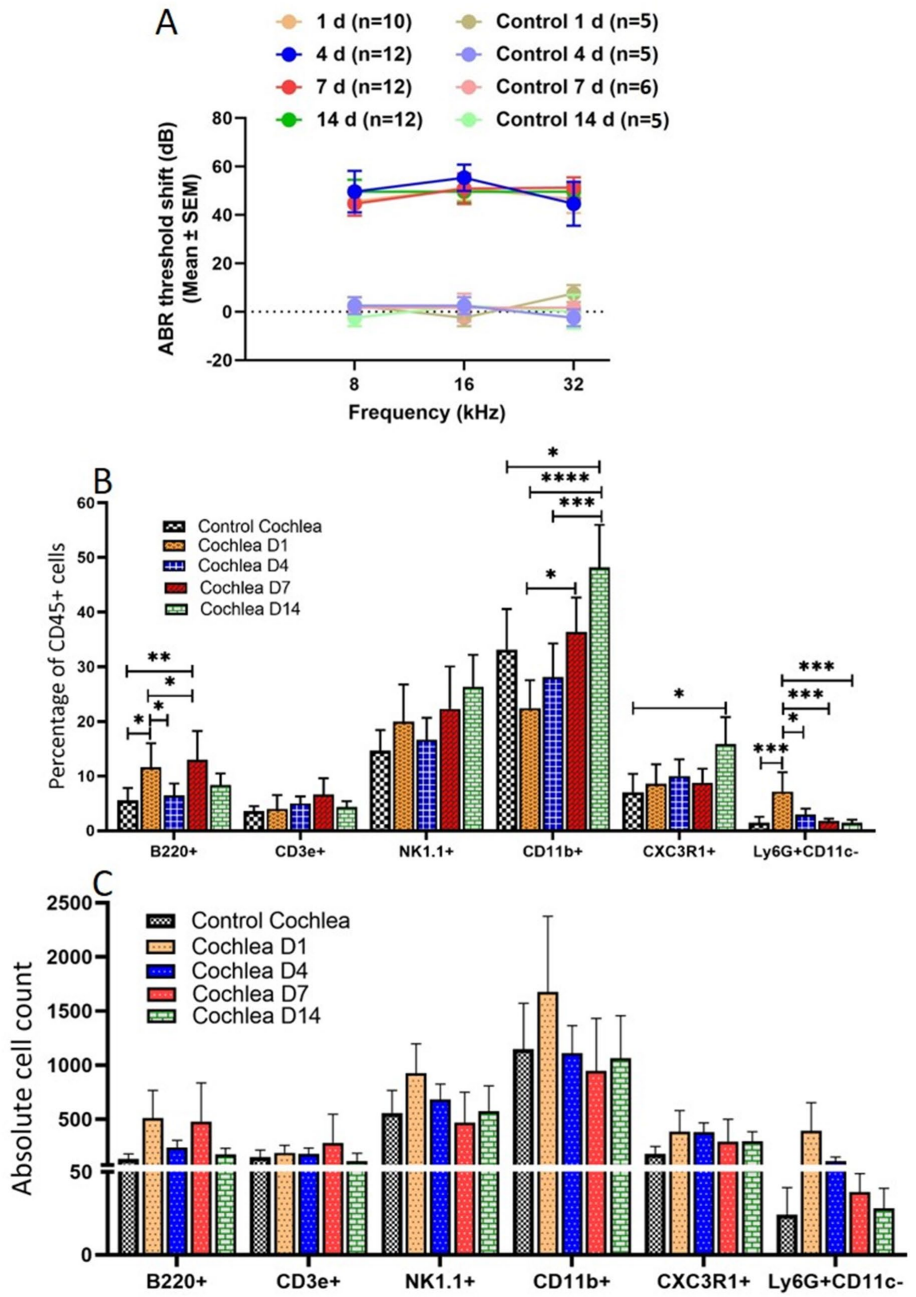
Auditory Brain Response (ABR) and flow cytometric evaluation of control and noise- exposed mice. The noise-exposed mice showed increased ABR threshold on days 1, 4, 7, and 14 as expected. There was no change in the ABR threshold in control mice (**A**). ABR threshold shifts at 8 kHz, 16 kHz, and 32 kHz after noise damage. Orange line: 1 day after noise damage, blue line: 4 days after noise damage; red line: 7 days after noise damage; green line: 14 days after noise damage. The numbers in parentheses in the figure indicate the number of mice tested. Noise trauma resulted in significantly increased population of immune cells in mice cochlea (**B**,**C**). Live CD45 + cells in the sample were analyzed for B, T, NK, and myeloid cells and myeloid cells were further gated for macrophages and neutrophils depending on their surface- markers as described in methods section. (**B**) Average percentage population of the B cells (CD45 + B220 +), T cells (CD45 + CD3e +), NK cells (CD45 + NK1.1 +), myeloid cells (CD45 + CD11b +), macrophages (CD45 + CD11b + CX3CR1 +), and neutrophils (CD45 + CD11b + Ly6G + CD11c −) from all mice (male + female together) in the experiment at day 1, day 4, day 7, and day 14 cochlea post noise exposure. B) Percentages of B220 +, CD3e +, CD11b +, CD11b + CX3CR1 +, and CD11b + Ly6G + CD11c− as part of the CD45 + population of immune cells in the control and noise-exposed cochleae. (**C**) Absolute counts of immune cells in the control and noise-exposed cochleae. Data are presented as mean ± SEM (n = 10, 12, 12, and 12 at day 1, 4, 7, and 14 respectively); *p < 0.05, **p < 0.01, ***p < 0.001; and ****p < 0.0001.

It has been shown that CD45 + cells in the cochlea increase in number after noise damage^6^. We sought to show how the composition of the CD45 + cells in the cochlea changed after noise damage. As both adaptive and innate cells are present in the cochlea, we focused on four time-points (1, 4, 7, and 14 days) after noise damage that would correlate with both adaptive and innate responses^30^. Previous studies have shown an increase in cell number and cytokine production at these time points^5,17^. After noise damage with 120 dB 8–16 kHz octave band noise for 2 h, we observed elevated ABR thresholds at 8 and 16 kHz, as expected, thereby confirming the hair-cell damage induced by the noise used in our study^4^. Stably increased ABR thresholds were also observed at 32 kHz from 1 to 14 days (Fig. 2A). Flow cytometry analysis of the noise- exposed cochlea showed heterogeneity of the immune cells at different time points (Supplementary Fig. 2). Supplementary Fig. 1 shows representative flow dot-plots illustrating the changes in the percentages of immune cells after noise damage after days 1, 4, 7, and 14; and, Supplementary Fig. 3 and Table 1 shows the percentages and the absolute counts of the immune cells at different time points after noise exposure in male and female cochleae analyzed separately. No significant difference in the immune cell populations of each sex was observed (Supplementary Fig. 3, Table 1). In the noise-exposed cochlea, compared to control cochleae, an increased proportion of the CD45 + population consisted of the following cell types was observed. An increase in the percentage of neutrophils was observed on day 1 (7.16 ± 3.54 vs 1.50 ± 1.03). 4 days after noise exposure, the percentage of macrophages was increased (10.03 ± 6.53 vs 7.05 ± 3.37); and after 7 days of noise exposure, T cells (6.65 ± 2.94 vs 3.56 ± 0.9) and NK cells (22.32 ± 7.61 vs 14.69 ± 3.73) were increased compared to control. An increase in the population of myeloid cells including macrophages (48.18 ± 7.80 vs 33.13 ± 7.44) was observed on day 14 (Table 2). An initial drop in the count of myeloid cells including macrophages (33.13 ± 7.44 vs 22.44 ± 5.11) was observed in our study (Table 2) which coincides with previous studies^31^. After a significant increase in the population of neutrophils at day 1 (10.03 ± 6.53) compared to control cochlea (7.05 ± 3.37) the population of neutrophils significantly decreased at day 4, 7, and 14 (Fig. 2B; Table 2). The results of flow-cytometry showed the highest number of immune cells on day 1 after noise exposure. The total percentages of adaptive immune cells (B and T cells) as part of the population in noise-exposed cochleae at day 7 was significantly increased compared to the control cochleae (Table 2). After the initial drop-off in percentage at day 1, the myeloid cell population significantly increased each subsequent time-point with its highest percentage at day 14 as compared to control cochleae (Table 2). Likewise, the macrophage population at day 14 was significantly increased compared to day 1, 4, and 7, and at day 4 compared to day 1 (Table 2). The increasing and decreasing trend in the percentages and absolute counts of different immune cells at different time points in noise-exposed cochlea compared to control cochlea is summarized in Fig. 2B,C. Among all immune cells, neutrophils (Day1), macrophages and monocytes (Day 14), and B cells (Day 4 and 7) (Table 2) showed significant changes in proportion of the total CD45 + population reflecting an initial response to damage by neutrophils followed by a responses by adaptive immune cells increasing from day 4 to day 7 then additional recruitment of myeloid cells by day 14. The estimates of the percentages of immune cell relative to macrophages population has been shown in Supplementary Table 2.

**Table 2:** Percentages of CD45 + immune cells in control and noise-exposed cochleae.

Immune cell	Control	Noise exposed				P values
		Day 1	Day 4	Day 7	Day 14	
	M + F	M + F	M + F	M + F	M + F	
Lymph + LiveCD45 +	6.53 ± 2.17	5.03 ± 0.91	7.35 ± 1.95	6.85 ± 1.95	12.15 ± 2.80 (*P = 0.01)	p = 0.00040 (D1 vs 14) p = 0.021 (D4 vs 14) p = 0.01 (D7 vs 14)
Lymph + LiveCD45 + B220 + (B cell)	5.59 ± 2.24	11.59 ± 4.40 (*p = 0.038)	6.41 ± 2.22	12.95 ± 5.29 (*p = 0.01)	8.38 ± 2.08	p = 0.05 (D1 vs4) p = 0.014 (D4 vs 7)
Lymph + LiveCD45 + CD3e + (T cell)	3.56 ± 0.92	3.97 ± 2.56	5.00 ± 1.27	6.65 ± 2.94	4.35 ± 1.05	NS
Lymph + LiveCD45 + NK1.1 + (NK cell)	14.69 ± 3.73	19.98 ± 6.79	16.64 ± 4.02	22.32 ± 7.69	26.38 ± 5.77	NS
Lymph + LiveCD45 + CD11b + (myeloid cell)	33.13 ± 7.44	22.44 ± 5.11	28.14 ± 6.12	36.32 ± 6.34	48.18 ± 7.80 (*p = 0.046)	p = 0.00007 (D1 vs 14) p = 0.05 (D1 vs 7) p = 0.001 (D4 vs 14)
Lymph + LiveCD45 + CD11b + CXC3R1 + (Macrophages)	7.05 ± 3.37	8.60 ± 3.55	10.03 ± 3.03	8.79 ± 2.56	15.86 ± 4.93 (*p = 0.024)	
Lymph + LiveCD45 + CD11b + Ly6G + CD11c-(neutrophils)	1.50 ± 1.03	7.16 ± 3.54 (*p = .001)	2.97 ± 1.06	1.78 ± 0.41	1.45 ± 0.55	p = 0.016 (D 1 vs 4) p = 0.001 (D1 vs 7) p = .001 (D1 vs 14)

Immune cells (B, T, NK, myeloid cells, macrophages, and neutrophils) population in the control and noise- exposed cochleae has been shown as the percentage population of the total CD45 + cells. Percentage population of CD45 + cells are from the total pool of live lymphocytes in the cochleae. Data are shown as mean ± SEM. A p value of < 0.05 was considered as significant. All the p values shown here are compared to control cochleae. *Control vs days. *M* male, *F* female, *NS* not significant.

### Immune cells in control and noise-exposed cochleae by immunostaining

**Figure 3:**
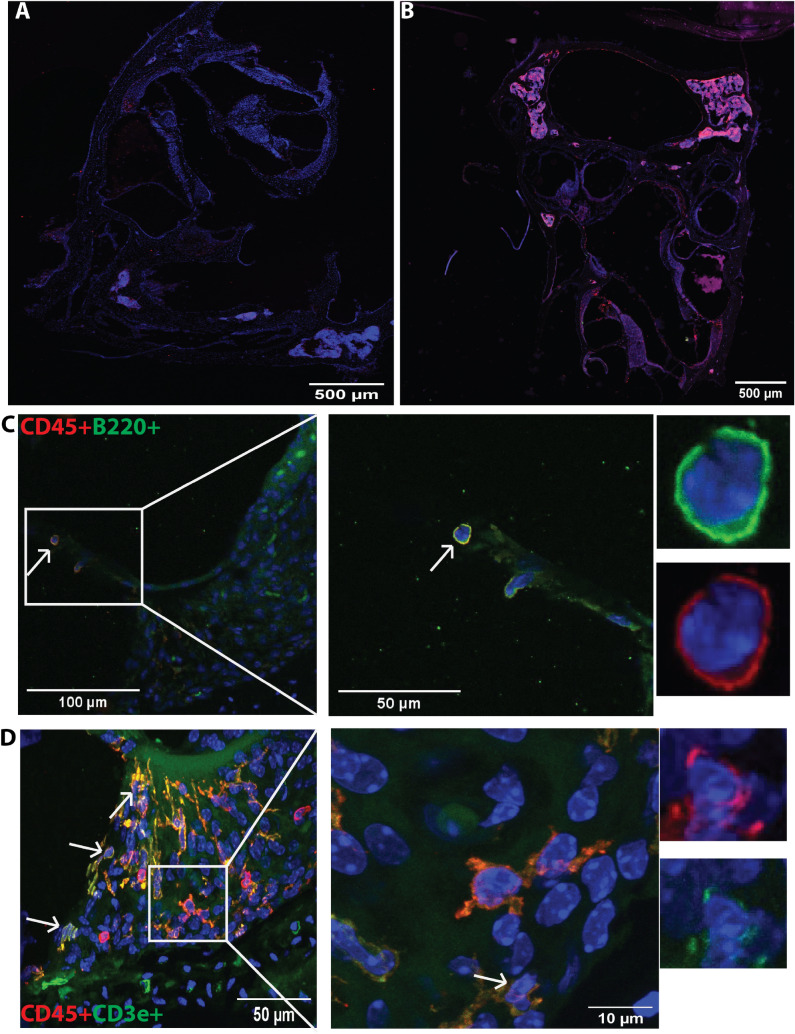
Immunofluorescence staining of the adaptive immune cells in the noise-exposed cochlea. The cochlea section stained with the marker of various immune cells showed positivity for CD45 (red), B220, and CD3e (green). (**A**) Control cochlea not exposed to noise, (**B**) noise-exposed cochlea at day 7, (**C**) CD45 + B220 + cells, and (**D**) CD45 + CD3e + cells in noise-exposed cochlea at day 7. The arrows show the presence of immune cells in the cochlea. In row C and D, first panel show the presence of immune cells (arrows) at 20X magnification, the second panel show the immune cells at higher magnification (× 40), and the last panel show individual cells for their morphology. These are the represented images (n = 4) for the presence of immune cells in the cochlea.

**Figure 4:**
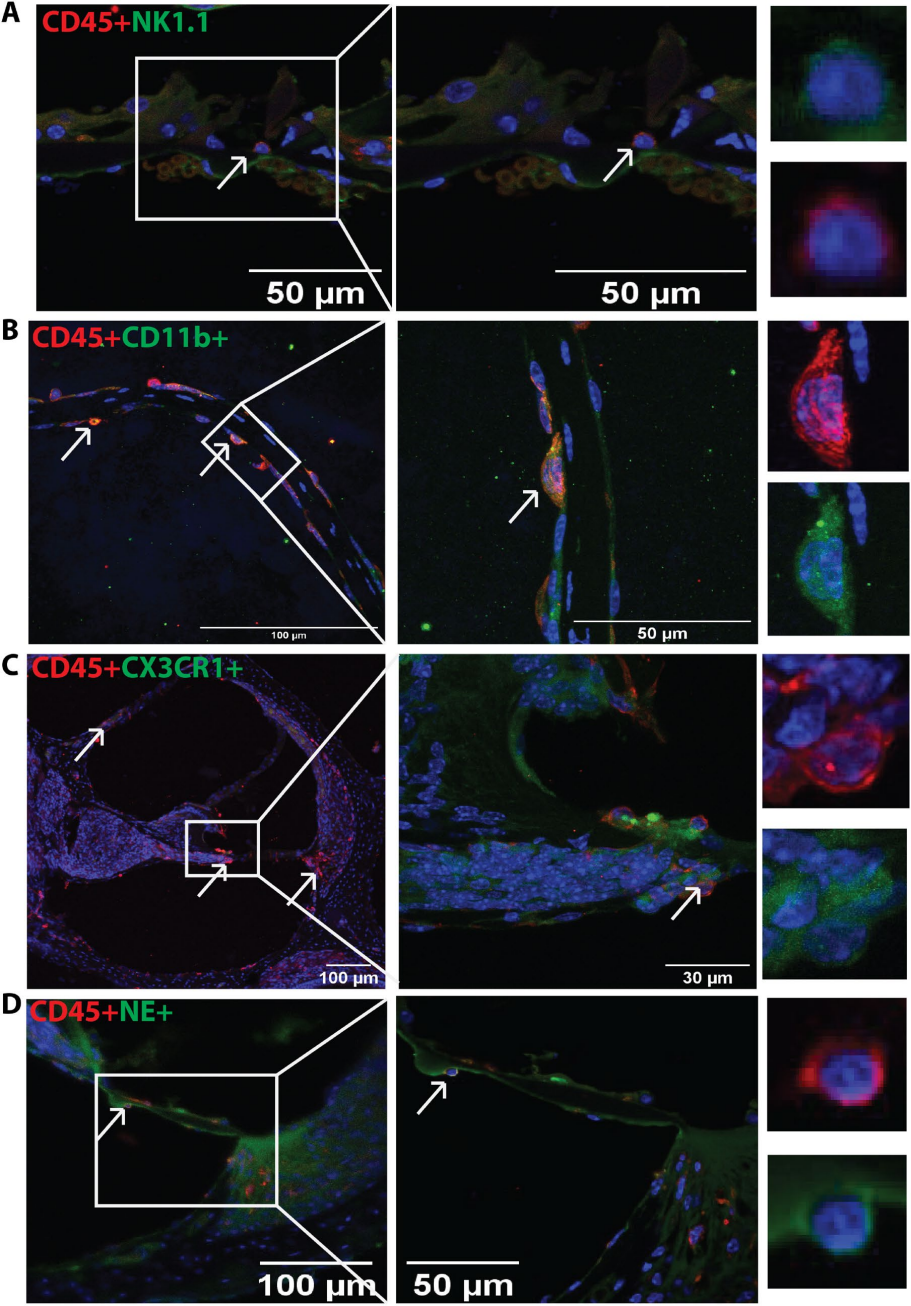
Immunofluorescence staining of the innate immune cells in the noise-exposed cochlea. The cochlea section stained with the marker of various immune cells showed positivity for CD45 (red), CD11b (red), CX3CR1, NK1.1, and neutrophil elastase (green). (**A**) CD45 + NK1.1 + cells, (**B**) Cd45 + CD11b + cells (**C**), CD45 + CX3CR1 cells and (**D**) CD45 + neutrophil elastase + cells in noise- exposed cochlea at day 7. The arrow shows the presence of immune cells in the cochlea. In rows A, B, C, and D, first panel show the presence of immune cells at × 20 magnification, second panel shows the immune cells at higher magnification (× 40), and the last panels show individual cells for their morphology. These are the represented images (n = 4) for the presence of immune cells in the cochlea.

**Figure 5:**
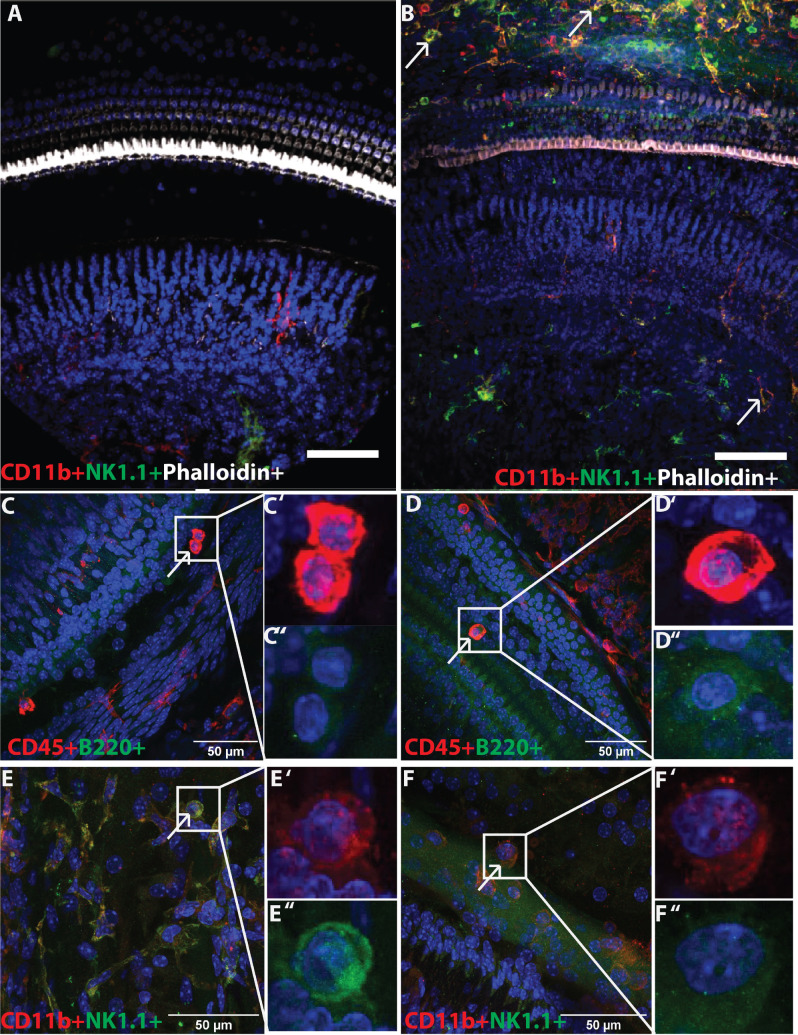
Immunofluorescence staining of the whole mount for immune cells. (**A**) control cochlea not exposed to noise, (**B**) Noise exposed cochlea at day 7, (**C**) Apical turn of the cochlea at day 7 post- noise exposure showing the immune cells (CD45 + ; red) and B cells (B220 + ; green), (**D**) Basal turn of the cochlea at day 7 post-noise exposure immune cells (CD45 + ; red) and B cells (B220 + ; green), (**E**) Apical turn of the cochlea at day 7 post-noise exposure showing the myeloid cells (CD11b + ; red) and NK cells (NK1.1 + ; green), (**F**) Basal turn of the cochlea at day 7 post-noise exposure showing the myeloid cells (CD11b + ; red) and NK cells (NK1.1 + ; green). C’, C”, D’, D”, E’, E”, F’, and F” show the individual images of the respective immune cell. These are the representative figures from 4 mice.

**Figure 6:**
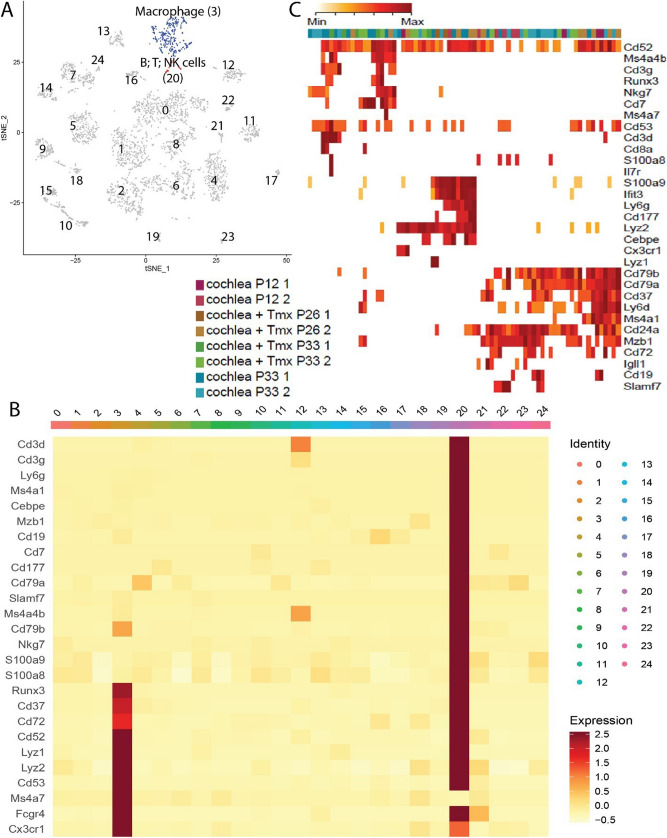
Single-cell RNA sequencing reveals a pattern of gene expression associated with several types of immune cells in the steady-state cochlea. (**A**) PCA followed by the t-SNE analysis of 5,470 cochlear cells from multiple mice. Distinct colors are used to define clusters of cells with similar gene expression. The identity of each cluster is based on the differential gene expression for that cluster. Cell clusters are labeled with the most differentially expressed gene for that cluster (Supplementary Fig. 6). (**B**) Heatmap of marker genes in the clusters shown in (**A**). The maximum value for each gene in log_2_(expected count + 1) (in red) and the minimum value for each gene in log_2_(expected count + 1) (in white) are shown as means. Each row represents a marker gene, and each column header indicates the corresponding color and number from (**A**). (**C**) Heatmap of selected marker genes from cluster 20, as defined in (**A**). The maximum and minimum values are shown in red and white, respectively, as in (**B**). The rows represent marker genes, and the columns represent individual cells within cluster 20. The color bar across the top indicates the condition of the cell in that column. The conditions are as follows: cochlea P12: cochlea at P12 with no tamoxifen induction; cochlea + Tmx P26: cochlea at P26 after tamoxifen induction at P12; cochlea + Tmx P33: cochlea at P33 after tamoxifen induction at P12; and cochlea P33: cochlea at P33 with no tamoxifen induction. The numbers after each condition indicate the replicate.

To verify the presence and the cochlear location of immune cell types, we next examined cochleae from control and noise-damaged mice, using confocal microscopy of whole-mount preparations of the sensory epithelium and mid-modiolar frozen sections of the entire temporal bone. Immunostaining of noise-damaged cochleae shows an increased population of immune cells in the noise-exposed cochlea compared to those of control animals (Fig. 3A,B). Stained cochleae from noise-exposed mice showed cells dual positive for CD45 + B220 +, and CD45 + CD3e + (Fig. 3), and dual positive for CD45 + and NK1.1, CD11b, CX3CR1, and neutrophil elastase (Fig. 4), suggesting the presence of immune cells. Cells showing positivity for CX3CR1 and neutrophil elastase were also positive for CD11b. Stained cryosections showed that immune cells were present mainly in the spiral ligament (T cells, NK1.1 cells, macrophages), scala tympani and scala vestibuli (mostly in mesenchymal lining and a few in epithelial lining; myeloid cells, T cells, NK1.1 cells, macrophages), basilar membrane (CD45 + B cells and neutrophils), the inner sulcus, and osseous spiral lamina (macrophages) (Figs. 3 and 4). The whole-mount staining showed the presence of immune cells that are underneath the basilar membrane and in the lateral wall of the cochlea (Fig. 5). The morphology of different immune cells was distinct. It should be noted that the B220 + cells were not CX3CR1 + and that their morphology was distinct (Figs. 3 and 4); the B220 + cells were round (Fig. 3 and Supplementary Figure 4), whereas the CX3CR1 + cells ranged in morphology from ramified to rounded (Fig. 4). These shapes mostly correlate to at-rest macrophages and activated macrophages, respectively^32^. The whole-mount immunofluorescence showed the presence of CD45 +, B220 +, CD11b + and NK1.1 + cells in the area between the spiral ligament and modiolus (Fig. 6). The presence of immune cells in immunostained noise-exposed cochleae supported the results of flow cytometry showing the presence of different immune cells, however, future studies are needed to gain a better understanding of the spatial pattern of distribution of immune cells in the cochleae.

### Single-cell RNA sequencing identifies a heterogeneous population of immune cells in the mouse cochlea

Single-cell RNA sequencing of mouse cochlear tissues revealed the expected cell types making up the inner-ear epithelium and neurons. Specifically, massively parallel single-cell RNA sequencing was used with droplet microfluidics (10X Genomics) and transcriptional profiles of rare cells isolated from the entire cochlea. Using this approach, 5% to 10% of the most highly expressed transcripts isolated from each cell can be quantified rapidly and accurately. We then acquired unbiased transcriptional profiles of cells present in the cochleae from the sensory epithelium and spiral ganglion but excluding the lateral wall and thereby identified immune cells^33,34^. Unexpectedly, our analysis detected gene expression corresponding to several types of immune cells, indicating that the resident immune cell population was more heterogeneous than previously thought. Figure 6A shows the 24 clusters of cells that could be statistically separated using PCA followed by t-SNE analysis. Clusters 3 and 20 are made up of immune cell types in the inner ear. The genes that differentiate these two groups from the other groups included in the analysis are shown in Fig. 6B. The genes that define clusters 20 and 3 encode a mix of surface markers, transcription factors, and functional proteins (Supplementary Fig. 5). Cluster 3 cells are marked by high expression levels of Cx3cr1, Fcgr4, Ms4a7, Cd53, and Lyz2. Of note, Cx3cr1 encodes the chemokine receptor for fractalkine expressed by monocytes, subsets of NK and dendritic cells, brain microglia and also by cochlear macrophages^6,11,35^, whereas Lyz2 encodes the protein lysozyme, which is a key component of the phagosome in myeloid cells^36^. These two genes alone mark this group as a candidate for cochlear macrophages. Figure 6C shows the genes that define cluster 20 (cluster 17 in Yamashita et al.^33^) in more detail. Cluster 20 is characterized by a large group of genes that include cell-surface markers for multiple immune cell types (Supplementary Fig. 6). Because the sample analyzed contained only a small number of cells, cluster 20 cannot be broken down into multiple subgroups on a statistical basis. However, it appears that three expression patterns exist within cluster 20. First, the genes Cd3g, Cd3d, Cd8a, Ms4a4b, Runx3, Nkg7, and Cd7 are all expressed by T cells^31,37,38^. The inclusion of Cd3d, Cd3g, and Cd8a here suggests that cluster 20 is at least partly made up of CD8 + cytotoxic T lymphocytes^31,37,38^. The next expression pattern is seen with Ly6g, Lyz2, and S100a9, all of which can be expressed together in granulocytes such as neutrophils^36,39–41^. The genes in the third block, namely Slamf7, Cd19, Cd72, Ly6d, Ms4a1, Cd37, Cd53, Cd24a, Mzb1, Cd79a, and Cd79b, are all associated with B cells^42–51^. Specifically, Cd79a and Cd79b encode the proteins required for the transmission of signals through the B-cell receptor, and Cd19 encodes a protein that acts as a co-receptor on the surface of the mature B cell^43,45,47,51^. The gene-expression clustering from the single- cell RNA-sequencing data revealed clusters of cells with patterns of gene expression corresponding to macrophages, neutrophils, T cells, B cells, and NK cells (Nkg7 subtype depending on the gene expression of Cd79a, Ms4a1, Cd79a, Nkg7, Cd52) in the mouse cochlea at P12, P26, and P33 (Fig. 6C)^33^.

## Discussion

The CD45 + cell compartment within the adult murine cochlea is composed of a heterogeneous mixture of immune cells. We observed B cells, T cells, CX3CR1 + macrophages, NK cells, and granulocytes (neutrophils), as previously reported^8^. However, we also observed an increased immune cell population after noise exposure (Fig. 2, Table 2). The increased number of B cells, T cells, macrophages, NK cells, and neutrophils in the noise-exposed cochlea compared to the control cochlea suggests the recruitment of immune cells post noise exposure either from cochlear resident pools or from peripheral circulation. Furthermore, the data presented here show that single-cell RNA sequencing, flow cytometry, and immunofluorescence are valuable and complementary methods of phenotyping immune cells in the adult cochlea at the mRNA and protein levels, respectively. Comparative analysis with control animals suggests that tamoxifen induction of Atoh1, Chrna9-EGFP, or other reporters (tdTomato)/Cre gene expression in supporting cells in our mouse models did not appear to affect the immune cell compartment^52,53^. Thus, existing scRNA-seq datasets from the cochlea may be analyzed to reveal valuable information about the homeostatic population of immune cells. The dissection parameters could even provide some positional information about the location of immune cell types. The transcriptional profiles identified through the RNA-sequencing analysis were further validated at the protein level by flow cytometry and immunostaining experiments. In addition, flow cytometry is a powerful tool that can enable the identification of additional surface and intracellular proteins. In future studies, these tools may provide insight into the function of immune cells in the cochlea after trauma.

The cell types identified in our studies here included B and T cells of the adaptive immune system, as evidenced by gene expression and phenotypic cell-surface protein expression. Cells of the innate immune system, such as macrophages, NK cells, and neutrophils, were also present. The expression of the Cx3cr1 and Ly6g genes and the proteins they encode indicates the presence of both neutrophil and macrophage populations. We found no evidence of NK cell-specific gene expression in the single-cell RNA-sequencing data, probably because of the limited sensitivity of the method; nonetheless, the detection of NK1.1 surface protein expression suggests that NK cells were present and increase after noise damage^25,26^. Increased number of NK cells in the cochlea after noise trauma in this study is supported by the notion that NK cells not only play a role in mediating cytotoxicity against a range of normal immune cells but also play a crucial role in controlling immune responses and maintaining homeostasis during inflammation^54^. This is also supported by the observation that NK cells are necessary for protection from CMV-induced hearing loss^55^. The composition of the CD45 + population in the cochlea shifted after noise damage, with an increase in B cells, T cells, NK cells, and myeloid cells were observed at 1, 4, 7, and 14 days after noise exposure (Fig. 2, Table 2). These data suggest that the immune response to noise damage is prolonged beyond the initial cell death and hearing loss. T cells regulate myeloid cells, such as macrophages, in their negative inflammatory response^56,57^. Thus, there may be a temporal window in which it is possible to intervene in the cochlea to promote tissue recovery and/or regeneration by manipulating the cytokine expression of T cells.

The migration of B220 + B cells into the organ of Corti after noise damage is especially interesting as it may indicate a hitherto unknown role for B cells in responding to the site of trauma. B cells can mature to make antibodies upon recognizing a specific antigen^49^. The presence of B cells in the cochlea in the absence or presence of noise damage is an interesting observation that may lead to new insights into the role of the immune system in the cochlea. However, the contamination of bone marrow and small capillaries during the preparation for flow cytometric analysis is possible. In the cochlea, most B cells were B220/CD45R + CD24 + CD19−, with only a few being CD19+. CD19 is a maturation marker for B cells; however, its expression is lost upon terminal differentiation of the cells into antibody-secreting plasma cells^49,58^. For future study, the inclusion of differential and specific markers for follicular, marginal, immature/transitional, naive, memory B cells, and plasma cells should be included to distinguish between migratory and resident or mature and immature B cells. Because CMV infects the inner ear, it is possible that B cells are present to eliminate viral infections^55^. This could have important implications for the application of gene therapy through viral delivery to hearing loss prevention and treatment. Further research is required to examine the B220 + population by lineage tracing and its role in the cochlea, especially after noise damage. In this study, we saw an increased number of T cells after noise trauma (Table 2). Previous studies have reported the role of T cell-mediated organ-specific autoimmune disorder of the inner ear in autoimmune sensorineural hearing loss^59^ and the presence of small number of CD4 + and CD8 + cells in the endolymphatic sac and occasionally in the cochlea, mostly in the peripheral region of Rosenthal's canal^60^. However, as to our knowledge, for the first time we are reporting increased population of T cells in the noise-exposed cochlea. The presence of T cells and their definitive role of T cells in the cochlea should be further explored.

The presence of resident macrophages and their recruitment after noise exposure has been discussed in the literature^10^. Resident macrophages may play an important role in protecting the inner ear via surveillance, scavenging, and tissue repair; however, the role of adaptive immune responses or infiltrating macrophages has not been firmly determined. The recruited macrophages may be detrimental to the inner ear and cause tissue damage by secretion of interferons, inflammatory cytokines, and chemokines or protective by secreting anti- inflammatory and reparative mediators^10,61,62^. In the noise-exposed cochleae, we found a significantly increased percentage of macrophages at day 14 and of myeloid cells at day 14 after an initial decrease at 1 day suggesting their recruitment in the cochlea (Table 2). Since we have not determined the phenotype of macrophages at different time points, elucidating the phenotype of macrophage in pro- and anti-inflammatory macrophages using corresponding markers and their phenotypic conversion with different time points using markers differentiating resident and migrated macrophages might be insightful in the determination of the functional role of the macrophage in the cochlea. We also cannot exclude the possibility that the increase in CX3CR1 + cells is not due to upregulation of the receptor on existing immune cells^63^. However, the overall increase in absolute cell number at the same time point (Day 14) continues to suggest increased recruitment.

The results of this study showed a significantly increased number of neutrophils on day 1 in noise-exposed cochlea compared to the control cochlea (Table 2). This finding is important because neutrophils, the most abundant leukocytes in the circulation, are the first line of defense in the innate immune system^64^. Neutrophils quickly respond to invading micro- organism and trap and kill the invading pathogens by phagocytosis, degranulation, and the release of nuclear material in the form of neutrophil extracellular traps. The multifaceted role of neutrophils in not only responding to micro-organisms but also playing a role in innate immunity by secreting cytokines, modulating the activities of neighboring cells, resolution of inflammation, regulating macrophages for long-term immune responses, and playing a role in innate immune memory has been discussed^65^. Further, the cytokines expressed by neutrophils and the heterogeneity of neutrophils has also been reported in the literature^66,67^. Our finding of significantly increased neutrophils on day 1 (acute inflammatory phase) suggests the protective role of neutrophils in the cochlea. Neutrophils have a heterogeneous phenotype and play a role in directing the innate immune; therefore, further analysis might be insightful in the cochlear immune response to damage. The increased presence of immune cells in the noise-exposed cochlea suggests studying the definitive role of these immune cells in cochlear inflammation might be helpful in developing novel therapeutics targeting the inflammatory cascade^68^.

## Conclusion

The presence of a heterogeneous composition of the immune cell populations in the adult cochlea and significantly increased number of CD45+ cells, comprised of T cells, NK cells, macrophage and neutrophils in the noise-exposed cochlea suggest the recruitment of innate and adaptive immune cells in the inner ear after noise trauma. This study has determined the population of various immune cells and their pattern of recruitment in the cochlea at various time points. Since these immune cells including T, B, NK cells, macrophages, and neutrophils play a crucial role in inflammation, controlling immune responses, and maintaining homeostasis, their presence in the cochlea suggests the ongoing inflammation after the noise trauma. This warrants the investigation of the definitive role of these immune cells in the cochlea. In the future studies, the use of differential and specific markers for immune cells should be included to differentiate between mature and immature or resident and migrating immune cells. Further, elucidating the level of various pro- and anti-inflammatory cytokines, chemokines and their course of change in the level will give an insight into the inflammatory aspect of the noise-exposed cochlea. Ultimately, this will elucidate whether inflammation is detrimental or protective to the inner ear and the window of time for intervention.

### Limitations and future directions

Non-availability of good antibodies to stain the immune cells in the mouse cochlea remained the major limitation of this study. This study has elucidated the heterogeneity of immune cells in the cochlea and their increased population after noise exposure. The flow cytometry data have been verified by immunostaining, however, the average number of immune cells on immunostaining has not been quantified. Future studies are warranted to elucidate the spatial orientation and absolute counts of the immune cells in each turn of the cochlea using reporter mice for immune cells. Additionally, we saw a decline of neutrophils after day one in flow cytometry, however, the immunostaining was done at 7 days post-noise exposure for all the cells because of the highest percentage for the immune cells at 7 days. Thus, flow cytometry and immunostaining of neutrophils at a different time points after noise exposure including zero to 24 h need future study to investigate their role in the noise-damaged cochlea. An interesting observation of the increased number of NK cells on flow cytometry is contrary to previous studies showing the highest number of macrophages in the cochlea. The higher number of NK cells on flow cytometry might be due to the mixed population of NKT cells, TCRalpha, TCRbeta, and NKB cells as we used only CD45 + NK1.1 + CD3e- for NK cell sorting. Further, NK cells might be NK1.1 + CD4 + T cells, NK1.1 + TCRαβ + cells and NK1.1 + CD8 + T, so detailed future studies are needed.

## Materials and methods

### Animal model

All animals were cared for in compliance with the guidelines of the St. Jude Children’s Research Hospital Animal Care and Use Committee, the NIH and Creighton University School of Medicine, Omaha, NE. The protocol was approved by the IACUC approving committee at St. Jude Children’s Research Hospital and Creighton University School of Medicine. The mice were fed Teklad 2018SC sterilizable diet with 18% protein and were kept at a temperature set point of 72 ± 2 ^°^F with a 12 h light and dark cycle. Single-cell RNA sequencing was performed on Fgfr3iCre +: Atoh1-HA: Chrna9-GFP: tdTomato + mice (129, FVB, and C57BL/6 J mixed background) at P12, P26, and P33. Four mice (eight cochleae) were treated with tamoxifen at 3 mg/40 g on 2 consecutive days at P12 and P13 to induce the ectopic expression of Atoh1-HA transgene driven by Fgfr3- iCreER-mediated CAG promoter in DCs and PCs to promote the conversion of DCs and PCs to HCs^33^. The rest of the experiments (ABR, flow cytometry, and confocal imaging) in the current study were performed on a colony of C57BL/6J mice that were purchased from The Jackson Laboratory. These mice are known to have age-related hearing loss^69^. Therefore, all hearing studies using these mice were completed before they reached 7 weeks of age to mitigate any contribution from the age-related hearing loss. Power analysis (effect size = 0.8, α = 0.05, power (1-β) = 0.80 with an allocation ratio of 1) was used to calculate the number of mice in each group. A total of 55 (28 males and 27 females) mice were used for these experiments. Mice were divided into control (n = 21) and noise-exposed group (n = 46). Control and noise-exposed group was further divided into three groups; 1 day (n = 5 and 10), 4 day (n = 5 and 12), 7 day (n = 6 and 12), and 14 day (n = 5 and 12) respectively, for control and noise exposed ABR readings and flow-cytometry. To rule out the bone-marrow contamination, 4 control mice with perfused cochlea were subjected to flow-cytometry analysis.

### Noise damage and auditory brainstem response

Noise damage was induced as previously described^70–72^ but with a few changes. ABR measurements were performed inside a soundproof booth (IAC Acoustics, IL). Animals were anesthetized with 2,2,2-tribromoethanol (375 µg/g bw, ip) and were exposed to an 8–16 kHz white noise band at 120 dB SPL for 2 h during the day. Subcutaneous needle electrodes were inserted behind the pinna (inverting), vertex of the skull (non-inverting) and base of the tail (ground). Tone bursts of 5 ms duration with 1.5 ms cosine-squared envelopes delivered at a rate of 21 stimuli per second with alternating polarity were generated using BioSigRZ software and RZ6 multi I/O processor system (Tucker-Davis Technologies, FL). Stimuli were presented as open field via a speaker (MF1; TDT, FL) placed 5 cm in front of the pinna of the animal. Evoked responses were amplified (20x), bandpass filtered (300–3,000 Hz) and average of 512 responses of 10 ms duration was recorded. Stimulus intensity was decreased in 5 dB increments, starting from 90 dB SPL to 0 dB SPL. Thresholds at 8, 16 and 32 kHz were identified by visual inspection from stacked waveforms as the lowest level at which reproducible response could be identified. Before the start of every session, stimulus presenting speaker (MF1) was calibrated with a ¼” microphone (PCB-378C10; PCB Piezotronics, NY) that was also place 5 cm in front of the speaker.

### Flow cytometry

A procedure for purifying immune cells from the mouse cochlea has been previously published^8^. In the present study, this protocol was used with slight modifications as follows. Cochleae from one adult mouse were dissected and pooled. The cochleae were further dissected such that the organ of Corti, lateral wall and modiolus were removed from the surrounding bone. Special care was taken to avoid opening the bone marrow cavity within the temporal bone. All the tissues were dissected out early in the morning around 8AM, processed for flow cytometry following the standard protocol, and given to the core facility by 2PM. All dissections took place in cold IMDM supplemented with 10% FBS. The cochlear tissue was collected by centrifugation and resuspended in Accutase (Innovative Cell Technologies, San Diego). The tissue was incubated at 37 °C for 10 min, with dissociation using a 1000 μL pipette tip at the 5-min mark and again at the end of the incubation. IMDM supplemented with 10% FBS was then added in an equal volume to the Accutase to quench the enzyme activity. Cochlear samples were collected once more by centrifugation, re-suspended, and stained with the antibody cocktail described below at a dilution determined by flow cytometry. After staining, the samples were washed once with IMDM then strained through a 40-μm cell strainer. As a positive control for immune markers, the spleen was also processed. The spleen was disrupted using the plunger from a 1 mL syringe. After being collected by centrifugation, the red blood cells in the sample were lysed and the spleen cells were likewise collected and washed again. The samples were stained and then strained through a 40-μm filter before being subjected to cytometry. Before the cochleae or spleen were stained, purified rat anti-mouse CD16/32 antibody (polyclonal, BD Pharmingen, Franklin Lakes, NJ) at a dilution of ≤ 1 μg/million cell in 100 μl was added to block the Fc receptors and prevent nonspecific binding of the antibodies. The antibodies used to stain immune cells were as follows: APC-Rat Anti-mouse CD45 (clone 30-F11, 559864, BD Pharmingen; 1:400), PE-CF594 Rat Anti-mouse CD45R/B220 (clone RA3-6B2, 562290, BD Pharmingen; 1:3,200), APC-Cy7 Hamster Anti-mouse CD3e (clone 145-2c11, 55596, BD Pharmingen; 1:800), BV605 Rat Anti-mouse Ly6G (clone RB6-8C5, 56299, BD Pharmingen; 1:200), BV510- Mouse Anti-mouse NK1.1 (clone PK136, 563096, BD Pharmingen; 1:200), PerCP- Cy5.5–conjugated anti-CD11c (clone HL3, 560584, BD Pharmingen; 1:800), FITC Rat Anti-mouse CD11b (clone M1/70, 11-0112-82, eBioscience, San Diego, CA; 1:200), PE-Cy7 Rat Anti-Mouse CD19 (clone 1D3, BD Pharmingen; 1:1,600), and Mouse CX3CR1 PE-conjugated Antibody (polyclonal, FAB5825P, R&D Systems, Minneapolis, MD; 1:200). LIVE/DEAD Fixable Violet Dead Cell Stain Kit (L34964, Thermo Fischer Scientific, USA) was used for sorting live cells. The absolute cell counts were obtained by using a Bio-Rad TC10 Cell Counter. 20 µl of the single-cell sample was mixed 1:1 with Trypan Blue Dye. The cells were counted by the machine with an output of the number of cells/mL and the number of live cells/ml. After running the full sample through the BD Aria Fusion, we took the percentages of each gate and cell type to calculate the number of individual cell types in the sample.

### Flow cytometry gating strategy and data analysis

A gate was first set from the forward- scatter versus side-scatter dot-plot of all events in the area that corresponded to the size and granularity of lymphocytes using a spleen as a reference. The cells in the lymphocyte gate were then plotted as forward scatter versus Live-Dead stain. The cells in the live gate were then examined in a plot comparing the forward-scatter width and side-scatter area; cells inside the gate were determined to be single cells (singlets). The cells within the singlet gate were then plotted as a histogram of CD45 expression. The live CD45 + cells from the cochlear cell sample were further analyzed for B cells (CD45 + B220+), T cells (CD45 + CD3e+), NK cells (CD45 + NK1.1+), and myeloid cells (CD45 + CD11b +) and the myeloid cells were analyzed for macrophages (CD45 + CD11b + CX3CR1+) and neutrophils (CD45 + CD11b + Ly6G + CD11c−). All flow cytometry was carried out on a Bio-Rad ZE5 Cell Analyzer, and the data were analyzed using FlowJo v10.6.1 (TreeStar).

### Immunostaining and confocal imaging

Whole-mount preparations were imaged after fixation with 4% paraformaldehyde and decalcification in 125 mM EDTA for 48 h. Decalcified cochlea was cryopreserved and 20-μm frozen mid-modiolar sections were immunolabeled for immune cells. The whole-mount preparations and sections were stained using the following antibodies at 1:50 to 1:100 dilution: goat anti-mouse CD45 (AF114, R&D Systems; a kind gift from Dr. Tejbeer Kaur), rat anti-mouse CD11b (MCA711, Bio-Rad), rat anti-mouse F4/80 (CI-A3-1: NB600-404SS, Novus Biologicals), rabbit monoclonal CD3e (SP7:SAB5500058, Sigma-Aldrich), rat anti-mouse CD45R/B220 (RA3- 6B2; 553083, BD Pharmingen), mouse monoclonal neutrophil elastase (NP57; sc-53388, Santa Cruz Biotechnology, Inc.), CD161/ NK1.1 Antibody (PK136; NB100-77528SS, Novus Biologicals), and rabbit polyclonal myosin VI (25-6791, Proteus BioSciences). The secondary antibodies used at a 1:1,000 dilution were Alexa Fluor 568–conjugated donkey anti-goat, Alexa Fluor 488– conjugated donkey anti-rat, Alexa Fluor 647 goat anti-rabbit (Invitrogen). Nuclei were stained with DAPI. Immunostaining was done using the standard protocol. Briefly, the sections were post- fixed for 10 min with ice-cold acetone and blocked with 10% horse serum in 0.1%Triton PBS solution for 1 h. The sections were incubated with primary antibodies overnight at 4C, washed three times for five minutes each with PBS followed by incubation for 1 h with the corresponding secondary antibody. The sections were washed again with PBS, stained with DAPI and mounted with Vectashield Mounting Media (H-1000). We also stained the cochlea with CD45 and rat anti-mouse CD31 (1:100 dilution) antibody to stain the capillaries and the presence of immune cells around them (Supplementary Fig. 7A,B). Images were obtained using a 40 × oil Apochromat objective on a Zeiss LSM 700 confocal microscope.

### Single-cell RNA sequencing

Single-cell RNA sequencing (scRNAseq) data^33^ was reanalyzed to elucidate the different types of immune cells and the pattern of gene expression associated with distinct immune cells in the stable cochlea. Cochlear tissue (organ of Corti and spiral ganglion) was dissected then incubated in an enzyme solution consisting of 50% Accutase (Innovative Cell Technologies, San Diego, CA), 0.02% trypsin (Thermo Fisher Scientific, Waltham, MA), and 125 µg/mL thermolysin (Sigma-Aldrich, St. Louis, MO) in Hank’s balanced salt solution (HBSS) at 37 °C for 3 min. Then, 0.02 mg/mL collagenase IV (Sigma-Aldrich) and dispase (Worthington Biochemical Corporation, Lakewood, NJ) were added to the enzyme solution and the tissue was incubated for a further 4 min at 37 °C. The tissue was then disrupted by trituration with a fire- polished glass Pasteur pipette. The sample was filtered through a 40-μm strainer to ensure a single-cell solution. After centrifugation at 500 × g for 5 min, the dissociated cells were resuspended in HBSS containing 0.5% fetal bovine serum (FBS), 0.04% bovine serum albumin, and 0.3 mM ethylendiaminetetraacetic acid (EDTA). The Chromium Single Cell 3′ v2 Reagent Kit (10X Genomics, Pleasanton, CA) was used to construct the library. Briefly, the sample of single cells was loaded onto the Chromium Controller instrument (10X Genomics) and the cells were individually encapsulated in oil and lysed. Reverse transcription was performed in each oil droplet before cDNA was amplified. A HiSeq 4000 system (Illumina, San Diego, CA) was used to perform next-generation sequencing. The Cell Ranger Pipeline (10X Genomics) was used to align the sequence reads to mouse reference genome mm10. Seurat 3.1 software was then used to analyze the gene-barcode matrices provided by the Cell Ranger Pipeline. Individual data sets with abnormally small or large library sizes were removed as they were suggestive of low-quality cells and doublets. Cell Cycle Scoring function was used to classify the cell-cycle stages; cells designated as being in G1 were chosen for further analysis. Expected counts were obtained by randomly generating numbers with binominal distribution, with the minimum numbers of unique molecular identifiers for all cells as a parameter size and the population of each gene as the probability to minimize the batch effect. The heterogeneity of cell cycle scores was then regressed out by ScaleData function. The scaled residuals represented a ‘corrected’ expression matrix, that can be used downstream for dimensional reduction. The MeanVarPlot function in the Seurat package was used to choose highly variable genes by calculating the average gene expression and dispersion for each with the default condition^73^. Seurat 3.1 in R was used to perform PCA followed by t-distributed stochastic neighbor embedding (t-SNE) analysis^73^. The FindAllMarkers function in the Seurat package (in the default condition) was used to identify differentially expressed genes. PCA and t-SNE analysis were further performed using differentially expressed genes to group cell types based on gene expression.

### Statistical analysis

Graph-pad prism (8.2.1) and IBM SPSS (version 26) software were used for analyzing the flow cytometry data. Univariate/Multivariate analysis of variance (SPSS v26) was used to evaluate the statistical significance between sexes, days of treatment, and the control and treatment group. For post hoc analysis, Tukey pairwise comparisons were used. There was no statistically significant interaction effect between sex and treatment groups on the combined dependent variables, F (28, 131) = 0.789, p = 0.764, Wilk’s Λ = 0.571, partial η^2^ = 0.131. Also, the simple main effect of sex was not statistically significant, F (7,36) = 1.677, p = 0.146, Wilk’s Λ = 0.754, partial η^2^ = 0.246. Since there was no significant difference between males and females, data were pooled for further analysis. All data have been presented as mean ± SEM. A p value < 0.05 (*p < 0.05, **p < 0.01, ***p < 0.001; and ****p < 0.0001) has been considered as significant.

## Supplementary information

Supplementary file 1
