# Supplementary material for: The immune response after noise damage in the cochlea is characterized by a heterogeneous mix of adaptive and innate immune cells
Source: Sci Rep. 2020 Sep 16;10:15167. doi: 10.1038/s41598-020-72181-6 (PMC7495466; doi:10.1038/s41598-020-72181-6)
Supplement: Supplementary file 1 [file 41598_2020_72181_MOESM1_ESM.pdf]

The immune response after noise damage in the cochlea is characterized by a heterogeneous mix of adaptive and innate immune cells

Vikrant Rai[1]*, Megan B. Wood[2]*, Hao Feng[1], Nathan. M. Schabla[3], Shu Tu[1], and Jian Zuo[1]

[1]Department of Biomedical Science, Creighton University School of Medicine, 2500 California Plaza, Omaha, NE 68178, USA

[2]Department of Otolaryngology-Head and Neck Surgery, Johns Hopkins University School of Medicine, 733 N Broadway, Baltimore, MD 21205, USA

[3]Department of Medical Microbiology and Immunology and Flow Cytometry Core, Creighton University School of Medicine, 2500 California Plaza, Omaha, NE 68178, USA

* These authors contributed equally

Corresponding Author

Jian Zuo

Dept. of Biomedical Sciences

Creighton University School of Medicine,

2500 California Plaza, Omaha, NE, 68178

Email: jianzuo@creighton.edu

Phone: (402)280-2916

Supp. Fig. 1. Quantitative analysis of immune cells in control spleen and perfused cochleae. Flow-cytometric analysis of perfused cochleae show patterns of expression similar to those seen in non-perfused mice. Panels C to H shows the representative dot-plots of CD45+ cells from the perfused cochlea (n=4). Panel I show the average percentage population and panel J shows the absolute count of immune cells in the perfused cochlea (male and female combined, n=4). The bars indicate the average percentage and absolute count of CD45+ cells. The numbers on the dot-plots shows the percentages of cells gated in the corresponding dot-plot. The numbers showed in the plots for B, T, NK, and myeloid cells are percentage of CD45+ cells while the numbers of macrophages and neutrophils are percentage of CD11b+ cells, whereas the number for all immune cells in panel I are shown as the percentage of CD45+ cells

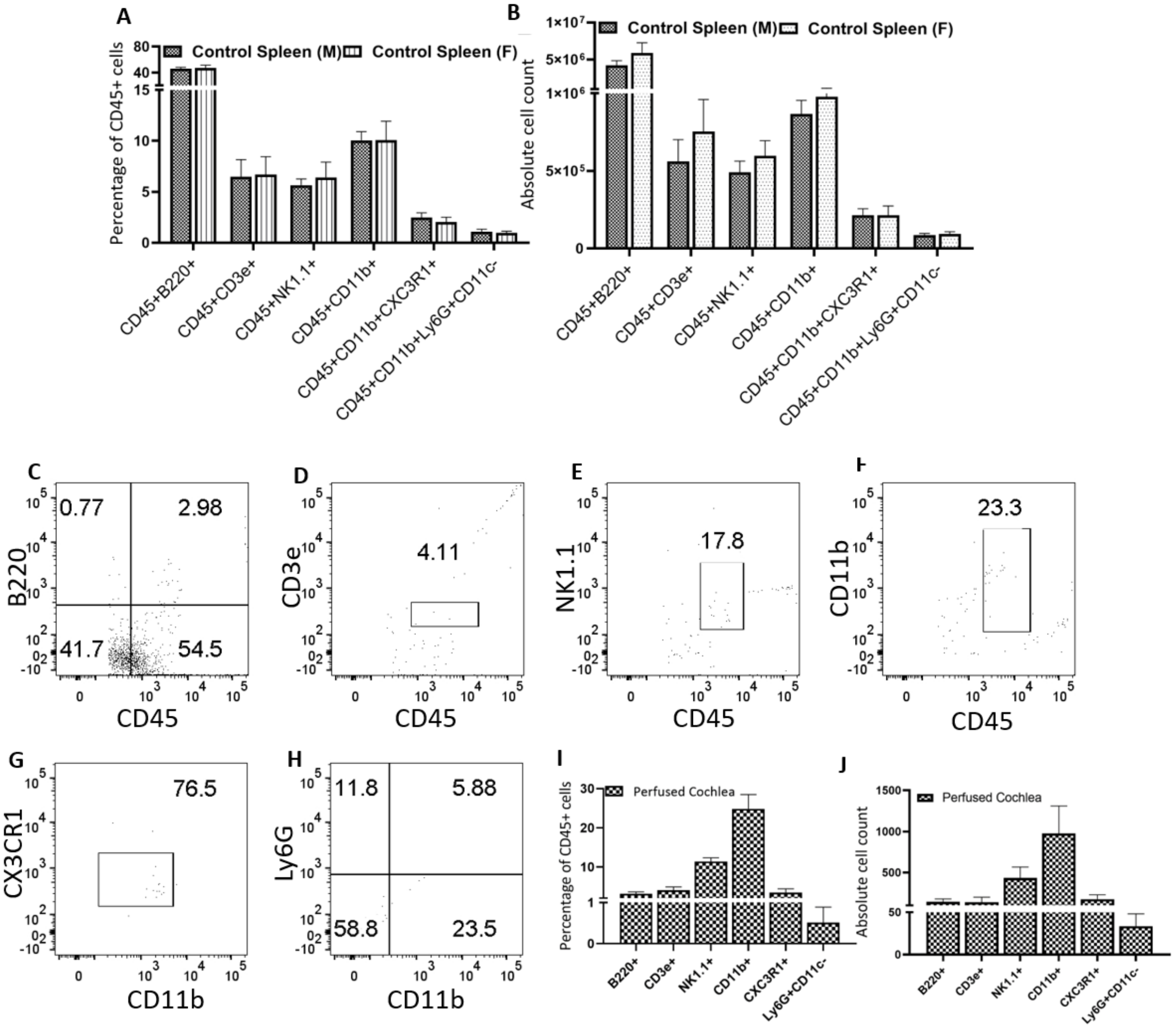

Supp. Fig. 2. Flow cytometry analysis of the CD45+ immune cells in noise-exposed cochlea. Live CD45+ cells in the sample were analyzed for B, T, NK, and myeloid cells and myeloid cells were further gated for macrophages and neutrophils depending on their surface-markers as described in methods section. Noise damage is accompanied by an increase in B cell, T-cell, NK- cell and myeloid-cell populations after noise exposure at day 1 (column 1), day 4 (column 2), day 7 (column 3), and day 14 (column 4). Flow dot-plots are the represented images of the percentages of the immune cells (CD45+), B cells (CD45+ B220+), T cells (CD45+ CD3e+), NK cells (CD45+ NK1.1+), myeloid cells (CD45+ CD11b+), macrophages (CD45+ CD11b+ CX3CR1+), and neutrophils (CD45+ CD11b+ Ly6G+ CD11c−) cells at various time points from all mice (n=10, 12, 12, and 12 at day 1, 4, 7, and 14 respectively) involved in the study. The numbers on the dot-plots shows the percentages of cells gated in the corresponding dot plot. The numbers showed in the plots for B, T, NK, and myeloid cells are percentage of CD45+ cells while the numbers of macrophages and neutrophils are percentage of CD11b+ cells.

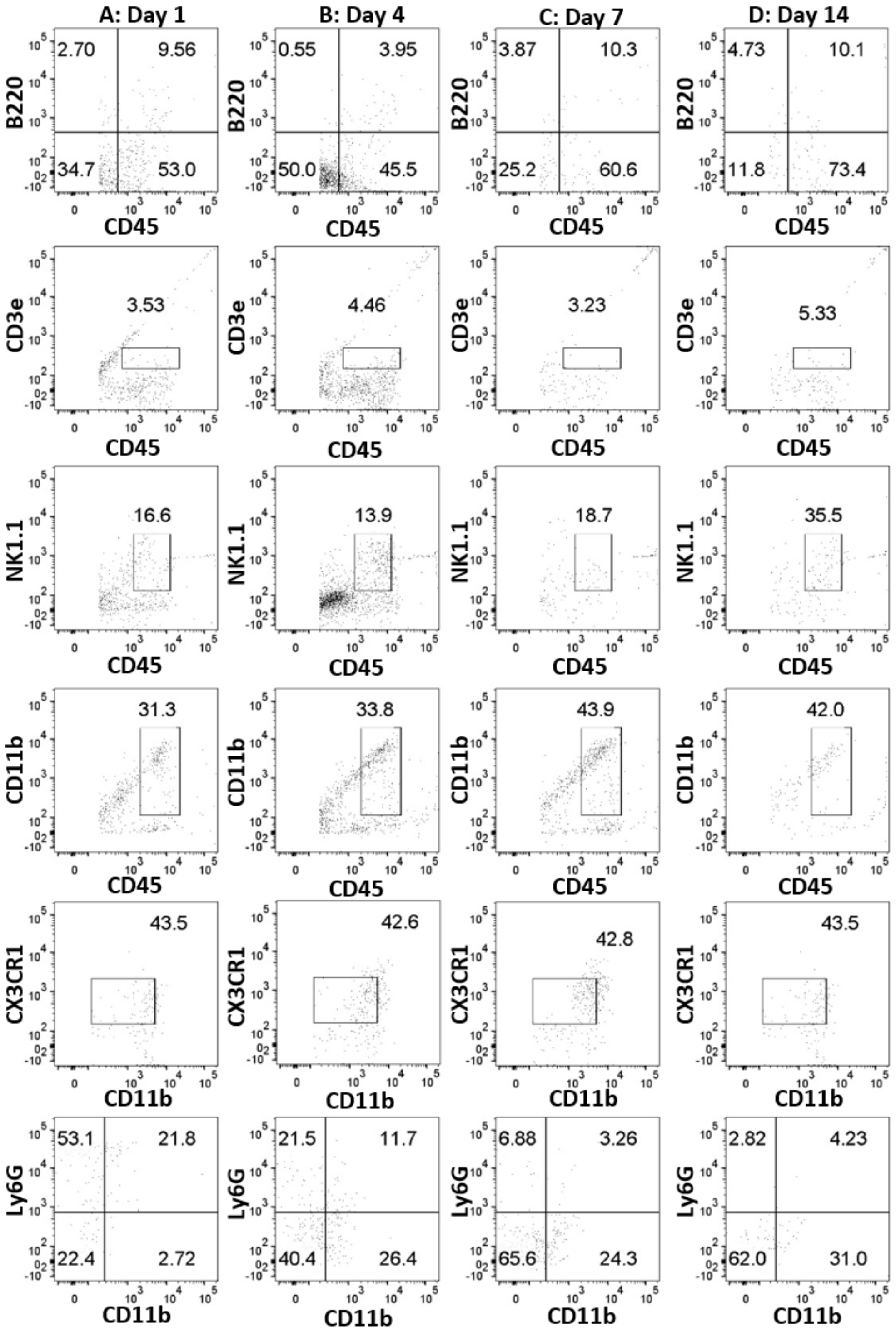

A: Day 1
B: Day 4
C: Day 7
D: Day 14
B220
CD45
2.70
9.56
34.7
53.0
0.55
3.95
50.0
45.5
3.87
10.3
25.2
60.6
4.73
10.1
11.8
73.4
CD3e
3.53
4.46
3.23
5.33
NK1.1
16.6
13.9
18.7
35.5
CD11b
31.3
33.8
43.9
42.0
CX3CR1
43.5
42.6
42.8
43.5
Ly6G
53.1
21.8
22.4
2.72
21.5
11.7
40.4
26.4
6.88
3.26
65.6
24.3
2.82
4.23
62.0
31.0

Supp. Fig. 3: Quantification of the percentages and absolute population of immune cells in noise-exposed cochlea. Live CD45+ cells in the sample were analyzed for B, T, NK, and myeloid cells and myeloid cells were further gated for macrophages and neutrophils depending on their surface markers as described in methods section. The bar graphs show the percentages and the absolute count(average ± SEM) of B cells(CD45+ B220+), T cells(CD45+ CD3e+), NK cells (CD45+ NK1.1+), myeloid cells (CD45+ CD11b+), macrophages (CD45+ CD11b+ CX3CR1+), and neutrophils (CD45+ CD11b+ Ly6G+ CD11c−) in the noise-exposed cochleae from all mice in the experiment at day 1 (A and B), day 4 (C and D), day 7 (E and F), and day 14 (G and H). Data are presented as mean ± SEM (n=10, 12, 12, and 12 at day 1, 4, 7, and 14 respectively). There was no significant difference in the immune cell population at different time points between two sexes. The height of the bar suggests the number of the immune cells as the percentage of CD45+ cells (panels A, C, E, and G) and the absolute counts of immune cells (panels B, D, F, and H) in the noise-exposed cochleae. M (male) and F (female)

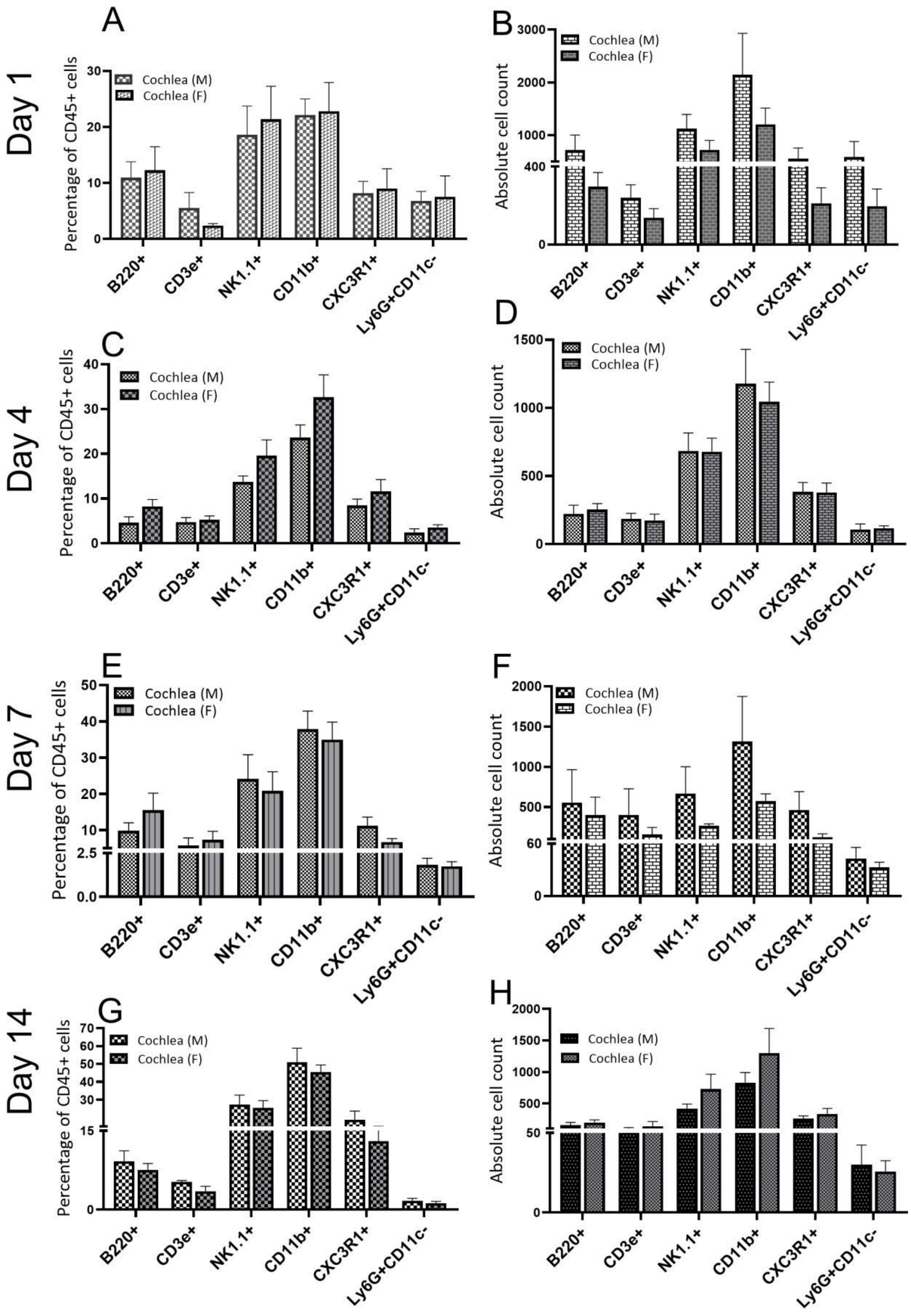
Day 1
Day 4
Day 7
Day 14
A
B
C
D
E
F
G
H
Percentage of CD45+ cells
Absolute cell count
Cochlea (M)
Cochlea (F)
B220+
CD3e+
NK1.1+
CD11b+
CXC3R1+
Ly6G+CD11c-

Supp. Fig. 4. Immunostaining shows the presence of B220+ cells in the cochlea and the organ of Corti 7 days after noise damage. Composite image of the spiral ganglion, organ of Corti, lateral wall, and bone marrow from unexposed (CTL) and noise-exposed (7day post damage) vibratome-sectioned cochleae. The inset (white box) shows the organ of Corti. Arrows indicate B220+ cells. Each B220+ cell is shown enlarged to highlight the round morphology associated with B cells.

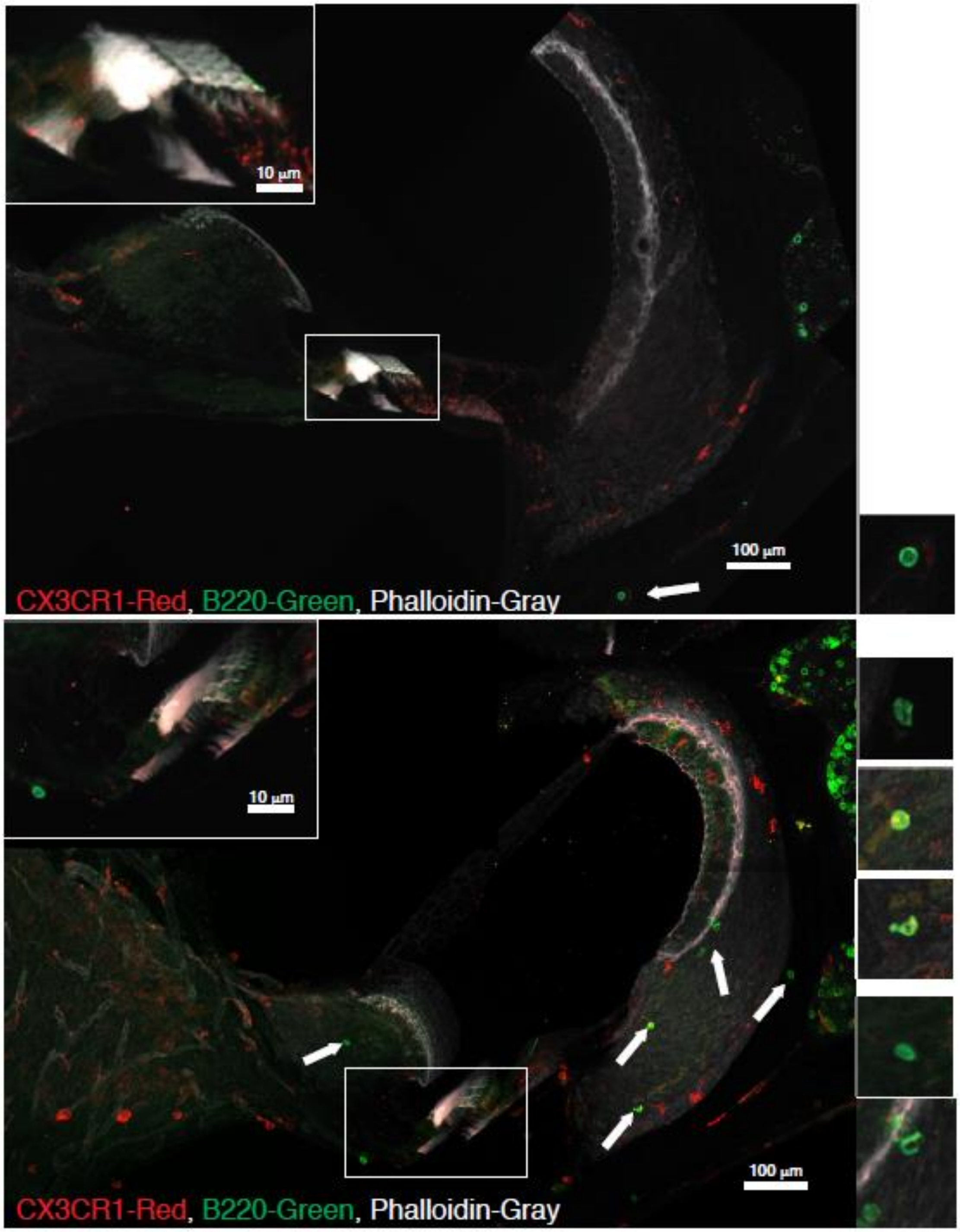

Supp. Fig.5: Gene expression tSNE plots of the immune cells used in the determination of heat map in Fig.6 B.

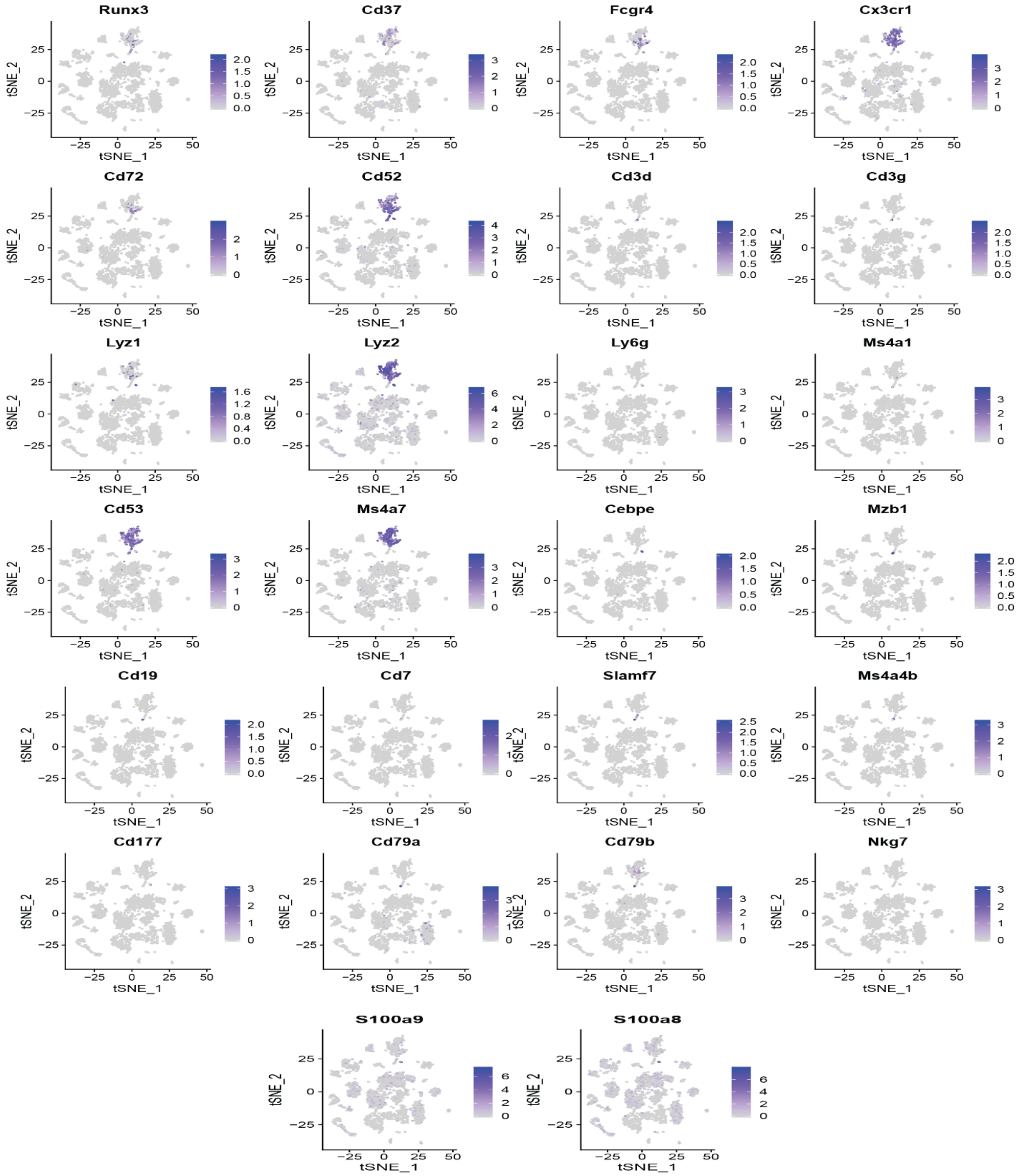

Supp. Fig. 6: Different clusters of the cell population in the scRNAseq derived tSNE plot in Fig.6A.

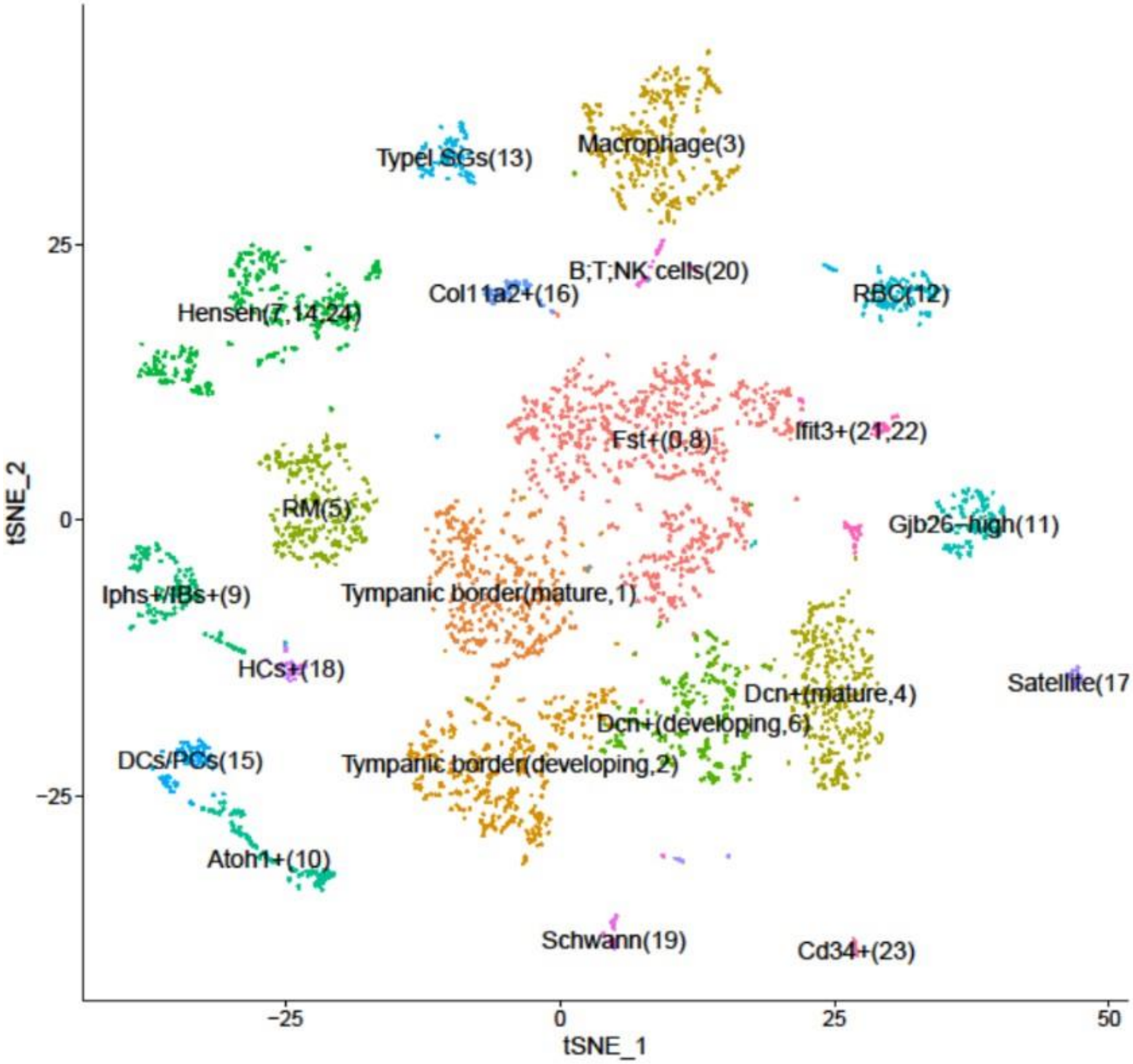

Supp. Fig.7: Immunostaining for small capillaries and immune cells in cochlea. Panel A shows the presence of immune cells (CD45) and small capillaries (CD31) in the lower spiral ligament region (arrow heads). Panels Aa and Ba’ (CD45+ immune cells); panels Ab and Bb’ (CD31+ endothelial cells lining the capillaries); panels Ac and Bc’ (DAPI), panels Ad and Bd’ (DIC images), and panels Ae and Be’ (composite images). The presence of empty lumen of the capillaries can be seen in the higher magnification (row B panel b’, arrowhead). Row C panels f and f’ shows the presence of immune cells (CD45+) in the control cochlea in spiral ganglion and lower spiral ligament region.

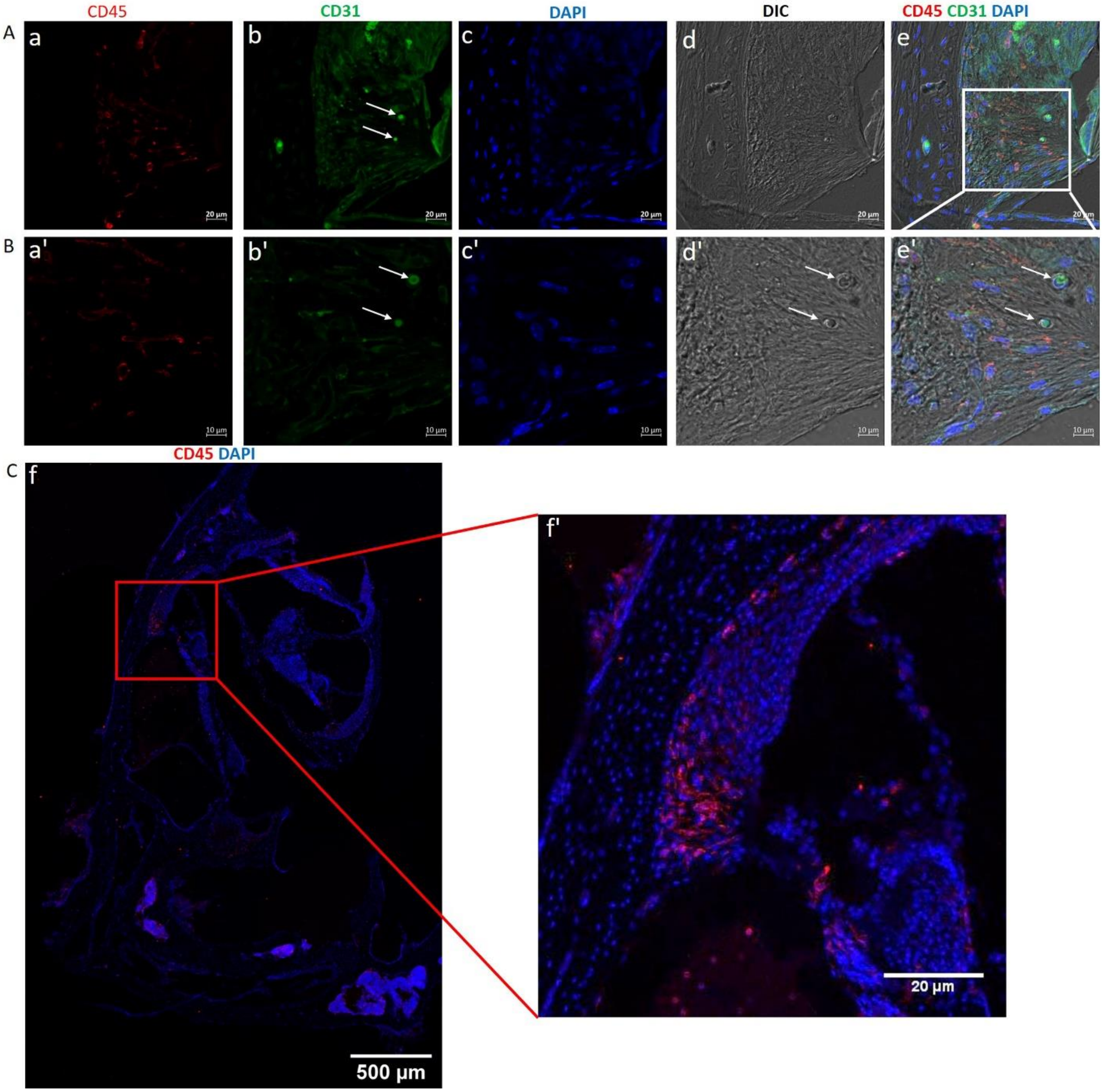

Supp. Table 1: Percentages of CD45+ immune cells in untreated and noise-exposed cochleae (male + female) at different time points. The data is shown as average ± SEM (n=10, 12, 12, 12 at days 1, 4, 7, and 14 respectively). There was no significant difference in the immune cell population at different time points between two sexes.

Immune Cell	Control		Noise exposed							
			1D post noise exposure		4D post noise exposure		7D post noise exposure		14D post noise exposure	
	Male	Female	Male	Female	Male	Female	Male	Female	Male	Female
CD45+	10.06 ± 0.51	3.71 ± 1.34	5.57 ± 0.87	4.49 ± 0.93	8.34 ± 2.18	6.35 ± 1.67	7.97 ± 2.05	5.91 ± 1.86	10.40 ± 2.79	13.90 ± 2.71
CD45+B220+ (B cell)	2.44 ± 1.02	8.11 ± 1.83	10.93 ± 3.69	12.25 ± 5.44	4.62 ± 1.83	8.20 ± 2.22	9.86 ± 2.84	15.53 ± 6.61	9.20 ± 2.59	7.56 ± 1.55
CD45+CD3e+ (T cell)	3.48 ± 0.84	3.63 ± 1.08	5.55 ± 3.54	2.38 ± 0.46	4.74 ± 1.44	5.27 ± 1.19	5.78 ± 2.71	7.37 ± 3.31	5.25 ± 0.37	3.45 ± 1.30
CD45+NK1.1+ (NK cell)	11.20 ± 1.12	17.48 ± 4.42	18.60 ± 6.62	21.36 ± 7.64	13.70 ± 1.91	19.58 ± 5.00	24.12 ± 8.66	20.80 ± 7.1	27.28 ± 6.84	25.48 ± 5.23
CD45+Cd11b+ (Myeloid cells)	24.40± 4.37	40.12 ± 7.11	22.10 ± 3.76	22.78 ± 6.67	23.62 ± 4.02	32.67 ± 7.07	37.92 ± 6.34	34.98 ± 6.82	50.94 ± 10.20	45.42 ± 5.14
CD45+ CD11b+ CX3CR1+ (Macrophage)	2.01 ± 0.93	11.08 ± 2.62	8.19 ± 2.71	9.02 ± 4.56	8.47 ± 1.99	11.59 ± 3.77	11.24 ± 3.07	6.76 ± 1.38	18.72 ± 6.38	13.01 ± 2.68
CD45+ CD11b+ Ly6G+ CD11c- (Neutrophils)	0.10 ± 0.06	2.62 ± 0.98	6.77 ± 2.21	7.54 ± 4.81	2.39 ± 1.22	3.56 ± 0.85	1.83 ± 0.49	1.75 ± 0.38	1.68 ± 0.63	1.21 ± 0.48

Supp. Table 2: Percentages of immune cell population relative to macrophages in the control and noise-exposed cochlea.

Immune cell	Control	Noise exposed			
		Day 1	Day 4	Day 7	Day 14
Macrophages	100	100	100	100	100
B cell	79	137	63.9	147	52
T cell	50	46	49.8	75	27
NK cell	200	232	165	253	166
Neutrophils	21	83	29.6	20	9
